# Pilot Study of Inclined Position and Infant Gastroesophageal Reflux Indicators

**DOI:** 10.1097/PG9.0000000000000312

**Published:** 2023-04-24

**Authors:** Ian M. Paul, Katherine E. Shedlock, Eric W. Schaefer, Ellen J. Stoute, Rachel Rosen

**Affiliations:** From the * Pediatrics, Penn State College of Medicine, Hershey, PA; † Public Health Sciences, Penn State College of Medicine, Hershey, PA; ‡ Aerodigestive Center, Division of Gastroenterology, Hepatology, and Nutrition, Boston Children’s Hospital, Boston, MA.

**Keywords:** gastroesophageal reflux disease, infant, hypoxia, bradycardia

## Abstract

To reduce gastroesophageal reflux, infants are commonly placed in an inclined position. We sought to observe the extent to which infants exhibit (1) oxygen desaturation and bradycardia in supine and inclined positions and (2) signs and symptoms of post-feed regurgitation in these positions.

**Study Design:**

Healthy infants aged 1–5 months with gastroesophageal reflux disease (GERD) (N = 25) and controls (N = 10) were enrolled into one post-feed observation. Infants were monitored in a prototype reclining device for consecutive 15-minute periods in supine position with head elevations of 0°, 10°, 18°, and 28° in random order. Continuous pulse oximetry assessed hypoxia (O_2_ saturation <94%) and bradycardia (heart rate <100). Regurgitation episodes and other symptoms were recorded. Mothers assessed comfort using an ordinal scale. Incident rate ratios were estimated using Poisson or negative binomial regression models.

**Results:**

Among infants with GERD, in each position, most had no episodes of hypoxia, bradycardia, or regurgitation. Overall, 17 (68%) infants had 80 episodes of hypoxia (median 20 seconds duration), 13 (54%) had 33 episodes of bradycardia (median 22 seconds duration), and 15 (60%) had 28 episodes of regurgitation. For all 3 outcomes, incident rate ratios were not significantly different between positions, and no differences were discovered for observed symptoms or infant comfort.

**Conclusions:**

Brief episodes of hypoxia and bradycardia as well as observed regurgitation are common for infants with GERD placed in the supine position after a feed with no differences in outcomes at various degrees of head elevation. These data may be used to power future, larger, and longer evaluations. ClinicalTrials.gov Identifier: NCT04542239.

**What Is Known**

- The American Academy of Pediatrics recommends that infants be placed supine for sleep to prevent sudden infant death syndrome.
- Infants with gastroesophageal reflex are often placed at an incline by caregivers as an attempt to reduce symptoms.

**What Is New**

- Brief episodes of hypoxia and bradycardia are common for infants aged 1 to <5 months with gastroesophageal reflux disease when monitored for 1 hour with pulse oximetry.
- Inclined positions between 10° and 28° were not significantly different than the supine position for hypoxia, bradycardia, observed regurgitation, or other symptoms associated with gastroesophageal reflux.

Since 1992, the American Academy of Pediatrics (AAP) has recommended that infants be placed supine for sleep on a flat, firm surface as part of a series of recommendations designed to prevent sudden infant death syndrome (SIDS) (1,2). SIDS deaths have decreased by 70% since the “Safe-to-Sleep” campaign (formerly “Back-to-Sleep”) was implemented that year. However, inclined sleep devices have been used by parents leading some infants to be placed to sleep in an inclined position, commonly between 10° and 30° (3), inclines explicitly not recommended by AAP. Additionally, infants spend considerable time in car safety seats (CSS) that keep infants at an incline, typically at 30°–45° (4), for prolonged periods, often while they are sleeping (5).

When using inclined sleep devices, parents were advised to always use restraints in the products and to discontinue use of those designed for sleep-purposes devices once an infant has the ability to roll over (6). Infant deaths have been reported in inclined sleep products when infants rolled into the prone position, with suffocation as the apparent cause of death (3). For CSS, it is also recommended that infants be restrained when using them; however, the AAP Policy Statement on Child Passenger Safety does not mention the recommended degree of incline for infants and young children (7).

Some caregivers independently incline their infants as a means to improve sleep, and placing an infant in an inclined position is sometimes recommended by healthcare providers or implemented by caregivers as a management strategy for infants with gastroesophageal reflux disease (GERD) (8). Although AAP recommends against, the 2016 SIDS guidelines cited only a single study with 24 infants in making the claim that inclined position does not reduce the symptoms of GERD (9). The 2022 guidelines cite a second study with 79 participants, where 30° in the prone position was found to reduce GER, although no benefit was found in the supine position (10). Notably, a study not cited in either guideline showed that reflux episodes were indeed reduced in infants placed at an incline (11), and inclined sleep has been shown to reduce GERD in adults (12). As such, we performed an exploratory study to add to the current evidence base regarding the effect of incline positioning on GER in infants. Infants with GERD and controls were assigned to a series of post-feed supine positions in random order to evaluate the extent to which infants exhibit (1) oxygen desaturation and bradycardia in supine and inclined positions and (2) clinical signs and symptoms of post-feed regurgitation in supine and inclined positions. We hypothesized that inclined positions would be associated with a reduced hypoxia and bradycardia. Further, we expected that increasing degree of incline would be associated with fewer witnessed episodes of regurgitation, fewer episodes of observed cyanosis, and less crying/fussing, hiccups, back arching, and breathing difficulty. With both objective measures and subjective assessments conducted during a 1-hour observation period, we anticipated that these findings will help clarify the role of inclined position in the management of symptoms typically associated with GERD.

## METHODS

### Study Design and Participants

A convenience sample of 25 infants with GERD and 10 controls aged 1 to <5 months were enrolled from one practice (Penn State Health, Hershey, PA) from March 2021 to January 2022. Inclusion criteria included gestational age at birth ≥34 weeks, weight ≥10th percentile, head circumference between the third and 97th percentile, and English-speaking parent ≥18 years old capable of consenting for participation. For infants with GERD, a healthcare provider diagnosis based on the clinical history and examination was required. Further, in the week before obtaining informed consent, the infant was required to have the following: (1) ≥4 visible daily spit-ups for at least 5 days in the week or (2) ≥2 daily spit-ups out of the nose for at least 5 days in the week or (3) score ≥16 on the Infant Gastroesophageal Reflux Questionnaire Revised (13). When applicable, parents agreed to not administer medication for GERD (eg, histamine H2 receptor antagonists) for 12 hours before participation. Exclusion criteria included signs of a more serious illness, history of a brief resolved unexplained event, or use of a home apnea monitor.

Before enrollment, infants were screened for eligibility including parental completion of the Infant Gastroesophageal Reflux Questionnaire Revised. Parents then completed the informed consent. This study was approved by the Penn State Institutional Review Board and registered at clinicaltrials.gov before participant enrollment.

**FIGURE 1.**
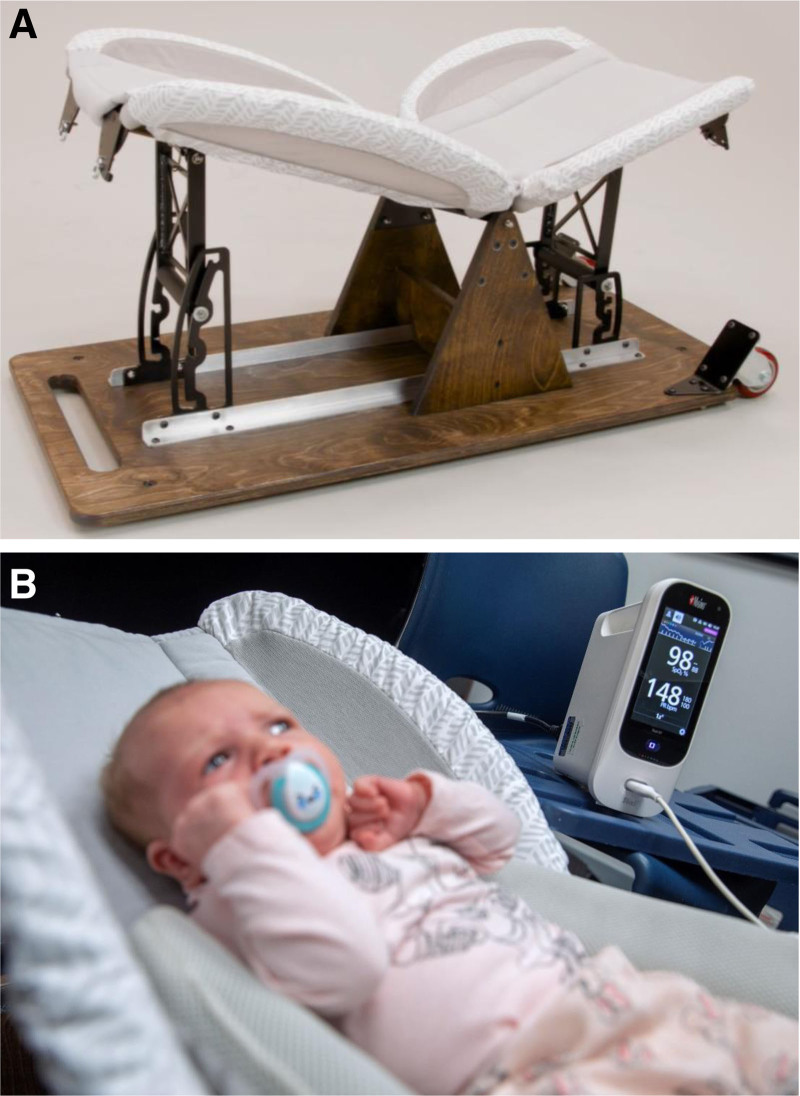
Infant reclining device: (A) side view; (B) with infant inclined on pulse oximetry.

At the study visit, parents fed their infant a typical liquid meal (ie, breastmilk or formula). Between 15 and 20 minutes after completion of the feed, infants were placed into an infant reclining device (Fig. 1) in a series of incline positions (0°, 10°, 18°, and 28°) for 15 minutes each in an order determined by a randomization list generated by the study statistician (E.W.S.). The device has a fully supported seat-back section and seat-bottom section. The infant’s back rested on the seat back and the legs were supported by the seat bottom. The back and bottom are independently adjustable and composed of a ridged, flat material. A removable, lightly padded covering encompassed the back and bottom for comfort. The fixture has lateral-containment panels primarily composed of mesh to ensure breathability. The inclined positions used for the study represent the range of incline of commercially available products (14). When the seat back was at a 0° incline, the bottom was also at 0°. When the seat back was inclined, the bottom was adjusted to 15° from the horizontal position.

During the observation period, the infant could be comforted with a pacifier, white noise, music, or other forms of distraction. Further, the parent was allowed to caress the infant. Following each 15-minute segment, a washout period was used such that the next segment did not begin for 1–5 minutes before the next position began so that each segment was not impacted by either the prior position or the change of position.

### Assessments and Outcome Measures

Demographic and chart data were collected, and a medical history was completed by parents, which contained information on sleep location, sleep positions, and diet (breastmilk or formula including use of cereal or milk thickeners). The Infant Gastroesophageal Reflux Questionnaire was completed by parents, who also gave a history of their child’s GERD onset and treatments to date.

At the study visit, the infant was monitored using the Masimo RAD-97 pulse oximeter (Irvine, CA) with data continuously collected regarding oxygen saturation and heart rate in 2-second intervals during each of the 4 segments (Supplemental Digital Content Figure 1, http://links.lww.com/PG9/A106). The oxygen saturation probe was attached to a big toe. For each segment, a study coordinator characterized whether reflux was seen (into mouth, out of mouth, out of nose, or repeated swallowing), the number of episodes as well as whether crying/fussing, hiccups, back arching, or breathing difficulty occurred. The coordinator also timed the duration of crying. For each segment, parents/guardians reported their perception of their infant’s level of comfort on an ordinal scale ranging from 1 (very comfortable) to 5 (very uncomfortable).

### Statistical Analysis

Our primary interest was infants with GERD for this study. Because our sample size was limited, we did not plan to compare the GERD and control groups directly. Instead, we conducted analyses separately for each group. Our intentions were to obtain preliminary data that characterized the relationship between incline position and outcomes.

For pulse oximeter data, hypoxia was defined as oxygen saturation <94% (15) and bradycardia was defined as heart rate <100 (16). The total number of episodes for an infant in a given incline setting was used in the statistical analysis.

The pulse oximeter was turned off between all positions as a washout period. To allow for the pulse oximeter to start up and track oxygen saturation and heart rate properly (run-in time), we excluded the first 20 seconds of each recording. We also applied this after any restart during an incline position, which occurred primarily when an infant kicked off the pulse oximeter. After manually reviewing all pulse oximeter data, we extended the run-in time in 6 other instances for an additional 2–8 seconds at which time the pulse oximeter began tracking properly. Finally, 2 infants had implausibly prolonged periods where the heart rate was recorded at <60 beats per minute at the beginning of a recording. These periods were excluded.

We fit statistical models only for infants with GERD. For the number of hypoxia and bradycardia episodes, we considered mixed effects Poisson and Negative Binomial regression models, both appropriate for count data. We used the Akaike’s Information Criterion (17) to determine that negative binomial regression fit the data best for both outcomes. The models contained a fixed effect for incline setting (dummy variables for each incline setting with 0° set as the reference group) and a random intercept effect for infant. The random effect accounted for the correlation among incline positions within the same infant. We reported the incidence rate ratio (IRR) and corresponding 95% confidence intervals (CIs) from these models, along with the predicted mean numbers of episodes.

The number of regurgitations was modeled using Poisson regression because it fit the data best according to the Akaike’s Information Criterion. Crying/fussing was recorded as binary (yes/no) and was modeled using mixed effects logistic regression with odds ratios reported. Maternal perception of comfort was modeled using mixed effects linear regression with mean differences reported. As above, all models included fixed effects for incline position and a random intercept effect for infant. Other rare outcomes were not modeled.

## RESULTS

### Participant Demographics and Baseline Variables

Participants with GERD (N = 25) were born at a median of 39.0 weeks gestation; 14 (56%) were female (Table 1). At enrollment, parents reported a median (range) score of 19.0 (8.0–30.0) on the Infant Gastroesophageal Reflux Questionnaire scale. Nearly all (88%) had ≥4 spit-ups per day from the mouth or nose for ≥5 days. Data for the 10 controls are listed in Table 1.

**TABLE 1.** Demographics and baseline variables of infants with GER and controls

	GER (N = 25)	Controls (N = 10)
Baseline and enrollment		
Sex, N (%)		
- Female	14 (56)	8 (80)
- Male	11 (44)	2 (20)
Race/ethnicity, N (%)		
- White, Non-Hispanic	22 (88)	10 (100)
- Black, Non-Hispanic	2 (8)	0 (0)
- Other	1 (4)	0 (0)
Gestational age in weeks at birth, median (range)	39.0 (36.1–41.4)	39.1 (37.0–40.7)
Birth weight in kg, mean (SD)	3.34 (0.44)	3.45 (0.39)
Maternal age in years, mean (SD)	32.7 (5.9)	31.1 (4.5)
Infant feeding mode, N (%)		
Breastmilk only	13 (52)	5 (50)
Breastmilk plus formula	6 (24)	1 (10)
Formula only	6 (24)	4 (40)
Milk thickener use, N (%)	2 (8)	1 (10)
IGER-Q* score, median (range)	19.0 (8.0–30.0)	4.5 (3.0–10.0)
≥4 spit-ups/day (mouth, nose) for ≥5 days, N (%)	22 (88)	0 (0)
≥2 spit-ups/day (nose) for ≥5 days, N (%)	1 (4)	0 (0)
Inclined sleep, N (%)	8 (32)	1 (10)
Child safety seat for sleep during night, N (%)	15 (60)	5 (50)
Prescribed medication for spit-ups/GER, N (%)	11 (44)	0 (0)
Over-the-counter and/or herbal supplements, N (%)	12 (48)	3 (30)
Study visit		
Age in months, median (range)	2.7 (1.2–4.8)	2.1 (1.3–4.9)
Weight in kg, mean (SD)	5.94 (1.24)	5.55 (1.17)
Study visit feed type, N (%)		
Breastfed	14 (56)	4 (40)
Pumped breastmilk	3 (12)	2 (20)
Formula	8 (32)	4 (40)
Duration of feed in minutes, mean (SD)	16.6 (5.7)	14.4 (5.6)

GER = gastroesophageal reflux.

*Infant Gastroesophageal Reflux Questionnaire–Revised.

At the time of the study visit, infants with GERD were a median of 2.7 months old. Roughly half (52%) were exclusively fed breastmilk. Use of a milk thickener was uncommon (8%), but nearly half (44%) were prescribed medication for GERD and a similar proportion (48%) took over-the-counter medications or herbal remedies for presumed gastrointestinal symptoms. Infants with GERD primarily slept in bassinets and cribs, although 8 (32%) slept at an incline and 15 (60%) spent time sleeping in CSS at night. Comorbid conditions were generally uncommon, although colic (16%), milk-protein allergy/formula intolerance (12%), constipation (8%), and eczema (8%) were reported. Controls were a median of 2.1 months old at the study visit, did not take prescribed medication, and did not have GERD comorbidities. Half of controls spent some portion of the night in CSS for sleep.

### Time in Each Position

Following feeds that lasted a mean of 16.6 (SD = 5.7) minutes, infants with GERD were placed on the infant reclining device in the position determined by the randomization scheme. All but one infant with GERD completed the study’s 15-minute assessments in all positions. For the one infant that withdrew, data from the 28° assessment was included as well as partial data from the 0° assessment that coincided with fussiness and study withdrawal. No data were collected at 10° and 18°. After accounting for the run-in time and periods where the pulse oximeter was not tracking, the mean time of observation in each position for infants with GERD ranged from 13.8 to 14.8 minutes (Table 2). All controls completed the study assessment in every position.

**TABLE 2.** Pulse oximetry recorded hypoxia and bradycardia, and observed regurgitation, crying and comfort in each position

	Infants with GER (N = 25*)				Controls (N = 10)			
	0°	10°	18°	28°	0°	10°	18°	28°
Analyzed observation time in minutes, mean (SD)	13.8 (1.8)	14.3 (2.1)	14.3 (1.6)	14.8 (0.5)	14.8 (0.1)	14.8 (0.3)	14.9 (0.1)	14.9 (0.2)
Hypoxia								
Episodes, N (%)								
0	16 (64)	16 (67)	15 (63)	19 (76)	7 (70)	8 (80)	5 (50)	9 (90)
1	3 (12)	5 (21)	4 (17)	4 (16)	0 (0)	0 (0)	2 (20)	0 (0)
2	1 (4)	2 (8)	4 (17)	1 (4)	1 (10)	1 (10)	1 (10)	0 (0)
≥3	5 (20)	1 (4)	1 (4)	1 (4)	2 (20)	1 (10)	2 (20)	1 (10)
Total seconds, median (IQR)†	24 (16–56)	8 (5–15)	30 (20–74)	12 (12–58)	84 (34–250)	66 (40–92)	16 (6–32)	714
Bradycardia								
Episodes, N (%)								
0	21 (84)	22 (92)	16 (67)	23 (92)	10 (100)	9 (90)	8 (80)	10 (100)
1	2 (8)	2 (8)	5 (21)	2 (8)	0 (0)	0 (0)	2 (20)	0 (0)
2	2 (8)	0 (0)	1 (4)	0 (0)	0 (0)	0 (0)	0 (0)	0 (0)
≥3	0 (0)	0 (0)	2 (8)	0 (0)	0 (0)	1 (10)	0 (0)	0 (0)
Total seconds, median (IQR)†	38 (11–56)	5 (2–8)	29 (14–38)	4 and 4	-	110	6 & 38	-
Regurgitation episodes, N (%)								
0	20 (80)	19 (79)	17 (71)	21 (84)	10 (100)	9 (90)	10 (100)	9 (90)
1	4 (16)	4 (17)	5 (21)	4 (16)	0 (0)	1 (10)	0 (0)	1 (10)
2	1 (4)	1 (4)	1 (4)	0 (0)	0 (0)	0 (0)	0 (0)	0 (0)
≥3	0 (0)	0 (0)	1 (4)	0 (0)	0 (0)	0 (0)	0 (0)	0 (0)
Crying/fussing								
Any episode, N (%)	9 (36)	7 (29)	13 (54)	12 (48)	4 (40)	3 (30)	4 (40)	1 (10)
Total minutes, median (IQR)†	2 (1–3)	5 (3–9)	3 (1–4)	2 (1–6)	2 (1–4)	4 (2–11)	2 (1–4)	1 (-)
Perception of comfort, mean (SD)	2.4 (1.2)	2.4 (1.4)	2.2 (1.2)	2.2 (1.1)	1.7 (1.3)	1.7 (1.0)	1.9 (1.0)	1.4 (0.5)

GER = gastroesophageal reflux; IQR = interquartile range.

*One participant withdrew after completing the 28° assessment, during the 0° assessment, and before beginning those at 10° and 18°.

†Participants with at least one episode; interquartile range (IQR) reported where applicable.

### Pulse Oximetry Recorded Apnea and Bradycardia

Among the 25 infants with GERD, 17 (68%) experienced ≥1 hypoxia episodes in at least one angle position. There was substantial variability within the sample as 8 participants did not have an episode, while one infant had 31 distinct episodes across the 4 positions. However, in each position, most infants (>50%) had no episodes (Table 2). Supplemental Digital Content Figure 2A, http://links.lww.com/PG9/A107 shows histograms of the number of hypoxic episodes for each angle. As shown in Table 3, the IRRs were lower in the inclined positions, although the 95% CIs were wide and overlapped 1.0. On the original scale, the fitted model corresponded to predicted mean numbers of 0.39, 0.20, 0.43, and 0.10 hypoxic episodes, respectively, by position. For infants with a hypoxic episode, the median times ranged from 8 seconds for 8 infants at 10° (interquartile range [IQR] 5–15 seconds) to 30 seconds (IQR 20–74 seconds) for 9 infants at 18°. Among 10 control infants, 6 (60%) had ≥1 hypoxic episode (Supplemental Digital Content Figure 3, http://links.lww.com/PG9/A108).

**TABLE 3.** Estimates of IRR, OR, or mean difference from fitted models for pulse oximetry recorded hypoxia and bradycardia, and observed regurgitation, crying and comfort

	Infants with GER (N = 25*)			
	0° (Reference group)	10°	18°	28°
Hypoxia IRR (95% CI)	1.0	0.56 (0.21-1.47)	0.72 (0.28-1.82)	0.59 (0.22-1.49)
Bradycardia IRR (95% CI)	1.0	0.35 (0.04-2.17)	3.99 (0.91-17.0)	0.33 (0.04-2.07)
Regurgitation IRR (95% CI)	1.0	1.03 (0.32-3.30)	2.06 (0.80-5.93)	0.67 (0.17-2.33)
Crying/fussing OR (95% CI)	1.0	0.69 (0.16-2.89)	3.12 (0.79-14.2)	2.05 (0.53-8.62)
Perception of comfort, mean difference (95% CI)	0.0	0.07 (−0.57 to 0.71)	−0.18 (−0.82 to 0.46)	−0.20 (−0.83 to 0.43)

CI = confidence interval; GER = gastroesophageal reflux; IRR = incident rate ratios; OR = odds ratios.

*One participant withdrew after completing the 28° assessment, during the 0° assessment, and before beginning those at 10° and 18°.

For bradycardia, 13 infants (54%) with GERD had ≥1 episode with 1 participant experiencing 13 distinct episodes at 18°. However, similar to hypoxia, in each position, most infants (>50%) had no episodes (Table 2). Supplemental Digital Content Figure 2B, http://links.lww.com/PG9/A107 shows histograms of the number of bradycardic episodes by position. The IRRs (95% CIs) ranged from 0.33 (0.04–2.07) at 28° to 3.99 (0.91–17.0) at 18°, both with reference to 0°, with wide confidence intervals (Table 3). On the original scale, the fitted model corresponded to predicted mean numbers of 0.24, 0.08, 0.96, and 0.08 bradycardic episodes, respectively, by position. Among infants with a bradycardia episode, median times ranged from 4 seconds for 1 infant with an episode at 18° to 28 seconds (IQR 11–56 seconds) for 4 infants with an episode at 0°. For control infants, bradycardia was rare.

### Observed Symptoms

Observed regurgitation for infants with GERD occurred in 16%–29% of infants in each position (Table 2). Supplemental Digital Content Figure 2C, http://links.lww.com/PG9/A107 shows histograms of the number of observed regurgitations by position. IRRs (95% CI) ranged from 0.67 (0.17–2.33) at 28° to 2.06 (0.80–5.93) at 18°, in reference to 0°. Among controls, only 2 episodes were observed. Crying/fussing episodes also occurred at a generally similar rate with odds ratios (95% CI) ranging from 0.69 (0.16–2.89) at 10° to 3.12 (0.79–14.2) at 18° in reference to 0°. Time spent crying was similar between positions, with medians ranging from 2 to 5 minutes. Other observed outcomes either rarely occurred or did not occur among those with and without GERD. For maternal perception of comfort among infants with GERD, mean differences (95% CIs) among incline positions were small ranging from −0.2 (−0.8 to 0.4) at 28° to 0.1 (−0.6 to 0.7) at 10°, in reference to 0°.

### Time Since Feeding and Outcomes

Due to the randomized order of the angle positions, the mean times since feeding were similar for the 4 positions (means of 44, 48, 42, and 46 minutes, respectively). Nevertheless, we fit models for episodes of hypoxia, bradycardia, and regurgitations that included time since feeding as an additional variable. In these models, the IRRs for angle positions were similar to models fit without the variable. Time since feeding was not associated with hypoxia or bradycardia episodes, but earlier times were associated with higher incidence of regurgitations (IRR = 1.59 [95% CI: 1.14–2.22] for every 15 minutes closer to feeding).

## DISCUSSION

This exploratory study demonstrates that generally brief episodes of hypoxia and bradycardia commonly occur for infants with GERD after feeding, and that these infants have visible regurgitation regardless of the angle of incline that they are positioned. We note that there were lower rates of hypoxia with inclined versus supine position and less bradycardia in the 10° and 28° positions, although this small, exploratory study did not achieve statistical significance for these outcomes. Degree of incline was not significantly associated with fewer witnessed episodes of regurgitation, less crying/fussing, or maternal perception of comfort, although all of these analyses were also limited by the sample size.

Prior evaluations of infants with GERD have examined the effect of inclined position (0° versus 30°) using a pH probe. Tobin et al (9) found that prone and left lateral positions reduced objective GERD compared with supine and right lateral position, but that incline had no effect on reflux. In contrast, Meyers et al (10) found 30° prone, but not 30° supine was beneficial, while Orenstein discovered less frequent postprandial GER for infants when placed in the prone position with head elevation compared with those without head elevation (11). Orenstein et al (18) also showed that a seated position with a 60° incline was associated with more GER than the prone position. Similar to Tobin, Loots et al (19) found the left lateral position reduced the number of impedance-detected reflux episodes, but this was not accompanied by a reduction in clinical symptoms including crying/irritability. Cardiorespiratory monitoring occurred in the study, although the data were not reported, and we believe ours is the first to report the data of such monitoring among infants with GERD. These studies are limited by their inability to detect nonacid reflux episodes and to provide the necessary granularity of reflux events to correlate with symptoms.

Parents of infants, with and without GERD, and their healthcare providers seek to improve infant comfort and their sleep. One concerning finding from this study was the common use of CSS for sleep by study participants, with >50% of study parents reporting use of CSS for sleep during the night. This is consistent with our prior report of unsafe sleep practices when mother-infant dyads are video-recorded overnight (20), and emphasizes the substantial importance of partnering sleep safety recommendations designed to prevent sudden unexpected infant deaths with those that help parents promote more effective sleep for their infants. Although our findings show possible reductions in hypoxia and bradycardia in the inclined position, the supine position did not achieve statistical significance, and they importantly add to the findings by Wang et al (21) that showed that inclined angles do not impact infant’s upper body muscle activity during supine lying.

This study has limitations. The sample size for this exploratory study was too small to arrive at definitive conclusions regarding differences between positions for our study outcomes. Further, the lack of objective measures of GERD (eg, pH-MII probe) limits our ability to correlate symptoms with episodes without visual regurgitation. Next, the 1-hour observation period may be insufficient to draw conclusions regarding the safety of each position, and the safety and degree of symptoms during prolonged periods of sleep was not a study goal. Another limitation is that feeding volume was not quantified as many of the participants were breastfed, and the impact of volume on outcomes cannot be determined. Additionally, our participants lacked demographic diversity, making the findings not generalizable to those from other backgrounds.

In conclusion, this exploratory study provides evidence of brief episodes or hypoxia and bradycardia at various degrees of incline for infants with GERD. Future evaluations may use these data to power a study to detect differences at various degrees of incline, particularly those that may evaluate infants for a longer study duration. Such data would support future guidelines regarding sleep safety and CSS use for infants with GERD.

## Supplementary Material
