# Supplementary material for: Pilot Study of Inclined Position and Infant Gastroesophageal Reflux Indicators
Source: JPGN Rep. 2023 Apr 24;4(2):e312. doi: 10.1097/PG9.0000000000000312 (PMC10187856; doi:10.1097/PG9.0000000000000312)
Supplement: Supplementary file 1 [file pg9-4-e312-s001.pdf]

Supplemental Figure 1. Example of Oxygen Saturation (SPO2) and Heart Rate (HR) Data Output from a Single Participant Monitored for 60 Minutes Across Four Positions*

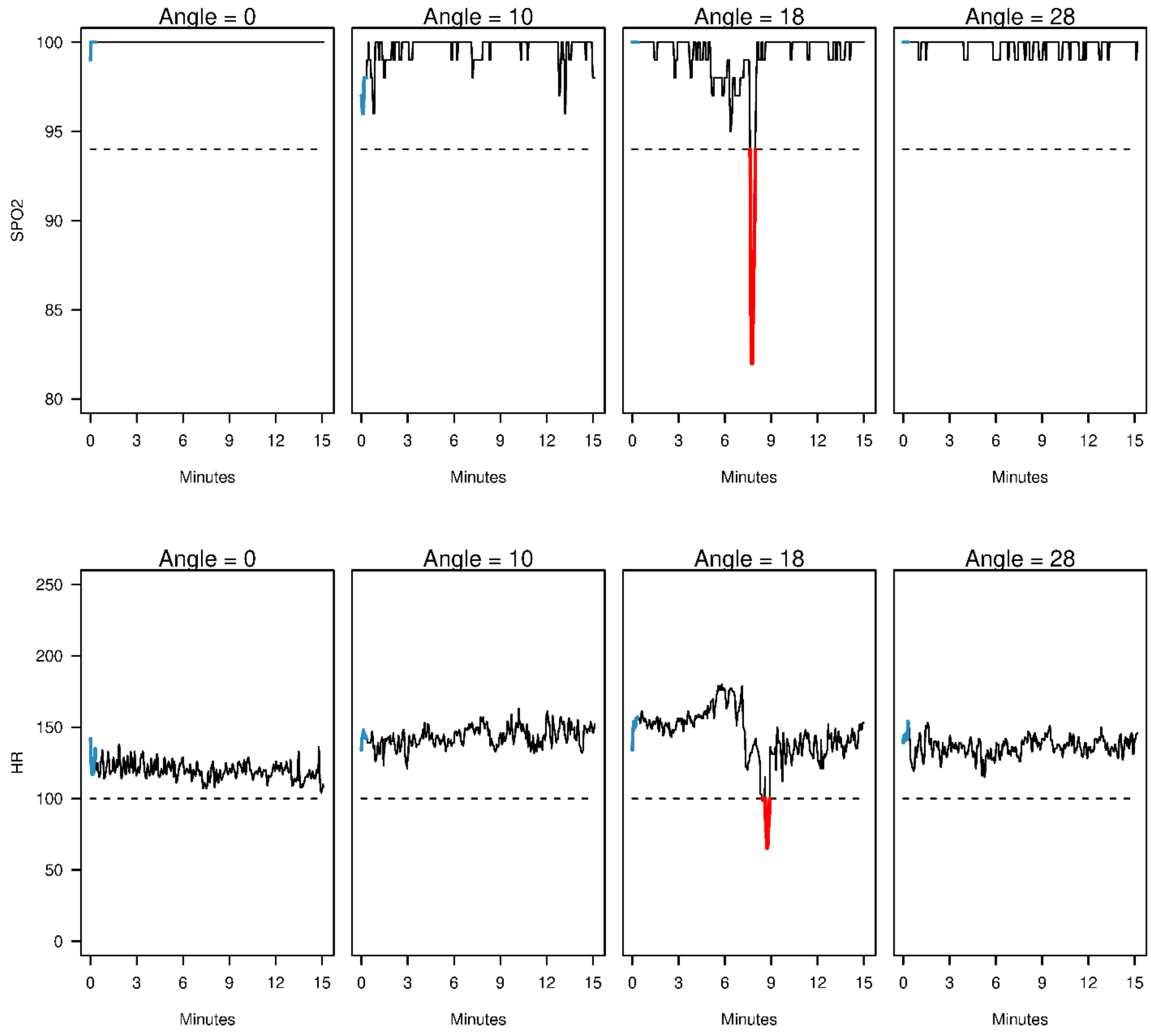

*blue color indicates data not included in analysis to allow for the pulse oximeter to properly track in each position; red color indicates time meeting criteria for hypoxia and bradycardia
