# Supplementary material for: Pilot Study of Inclined Position and Infant Gastroesophageal Reflux Indicators
Source: JPGN Rep. 2023 Apr 24;4(2):e312. doi: 10.1097/PG9.0000000000000312 (PMC10187856; doi:10.1097/PG9.0000000000000312)

Supplemental Figure 3. Histograms of the number of hypoxia, bradycardia, and regurgitation episodes at each incline angle for control infants (N=10)

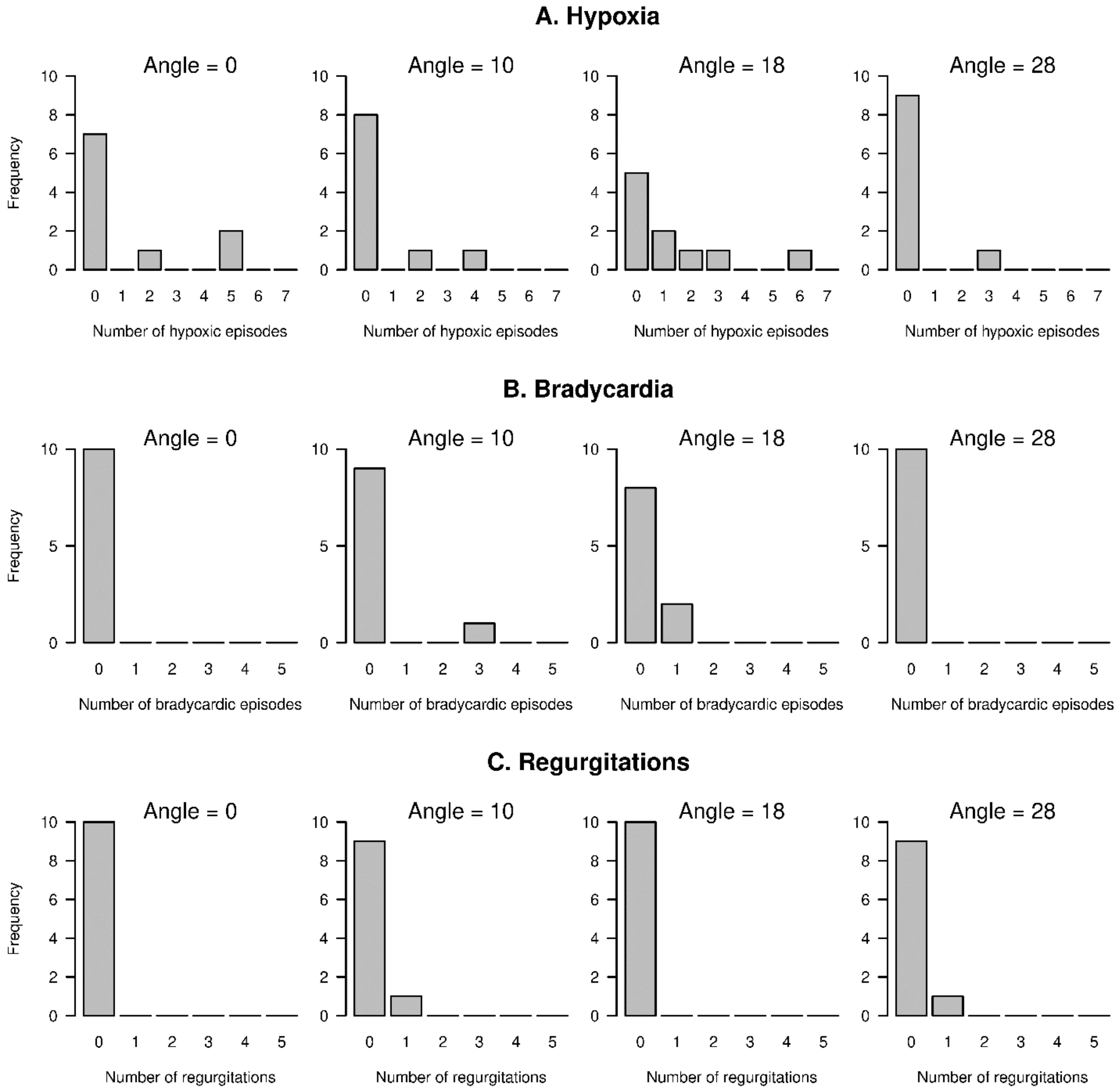

Supplement: Supplementary file 3 [file pg9-4-e312-s003.pdf]
